# The financing of stand-alone palliative Care Services in Uganda: analysis of the implications for sustainability

**DOI:** 10.1186/s12904-019-0434-5

**Published:** 2019-06-05

**Authors:** Jacinto Amandua, Melkizedeki Stephen Kimaro, Eddie Mwebesa, Ivan Mugisha Taremwa, Christine Atuhairwe

**Affiliations:** 1 Hospice Africa Uganda, P.O Box 7757, Kampala, Uganda; 2 East and Southern African Management Institute, P.O Box 3030, Arusha, Tanzania; 3 Clarke International University, P.O Box 7782, Kampala, Uganda

**Keywords:** Palliative care, Sustainability, Stand-alone, Uganda

## Abstract

**Background:**

Sustainable funding is key for ensuring the quality and coverage of palliative care services. This study examined the sources of funding for stand-alone palliative care services in Uganda as well as their services financial sustainability plans.

**Methods:**

Researchers conducted a cross sectional survey of all stand-alone palliative care organizations that have operated for five or more years. Researchers administered a questionnaire survey and interviews on the audited financial statements, services provided and sustainability plans.

**Results:**

Nine of the stand-alone palliative care organizations surveyed had operated for five to 25 years. 93% of the funding for palliative care services comes from donations; while 7% is from income generating activities. 94% of the donations are from external sources. The Government of Uganda’s major contribution is in the form of medicines, training and payment of taxes. All the organizations had good financial records. Six of the fifteen Hospices/palliative care providers had sustainability plans included in their operational manuals. The older organizations (those that had been operational for more than 10 years) had better resource mobilization capacity and strategies.

**Conclusion:**

The majority of stand-alone palliative care organizations in Uganda are largely donor funded. They have considerable financial sustainability and fund-raising capacity. Government support is in the form of medicines and training. Based on this study findings, the capacity of the stand-alone palliative care services to raise funds should be increased. The Government of Uganda should include palliative care in the national health system and increase funding for these services.

## Background

The incidence of cancer, and the associated risk of new cases remains high, and the number is predicted to double by 2030 [1, 2]. World Health Organization (WHO) estimates that 9100 males and 9000 females die of cancer annually, 64% of whom would have benefited from palliative care [3]. Patients with cancer experience severe pain in their course of illness especially as cancer progresses [3]. Consequently, the need for palliative care is evident, especially in Africa as the risk of cancer is at its peak [3, 4]. While palliative care is desired, access to suitable holistic palliative care (including effective pain management) is often unavailable in low-income countries [5]. This was confirmed by a survey conducted by the African Palliative Care Association (APCA) in 2003 which found that 45% of African countries had no identifiable hospice or palliative care activity, and only 9% can be classified as having services that have been integrated with mainstream health provision [6]. In Uganda, palliative care is not universally available to patients and families who need it and receives paltry governmental funding with only 69 out of 112 districts providing palliative care through both government and non-governmental organization health (NGO) facilities. Cancer and HIV/AIDS form the biggest proportion of patients in need of palliative care in Africa [5, 6]. Though most patients are cared for at home, there is limited access to palliative care services especially pain and symptom control and supportive services at the community and health unit level [7]. Many patients in low-income countries are in need of financial and material assistance alongside their medication in an environment where health workers and care givers are often stressed and over worked [6]. The establishment of Hospice Africa Uganda introduced palliative cares services to Uganda. Hospice Africa Uganda has served as a model for the development of hospice and palliative care services in Uganda and Africa [8]. The number of people in need of palliative care in Uganda is on the rise for example, an approximated 120,000 people are living with AIDS, and an estimated 90,000 have cancer with about 35,000 new cancer patients diagnosed yearly [3]. Specialist palliative care is available in all the national and regional referral hospitals, and most NGO and general hospitals. There is need to scale up service provision, training, and mainstreaming palliative care into the health system. Through policy improvement, drug availability, education and an integrated service delivery improved access and sustainability will be ensured [3, 9]. Further, despite the high incidence of cancer, the availability of morphine in low-income countries remains a challenge, with only three African countries (Uganda, South Africa and Tanzania) having palliative care integrated into their national health policies and strategies, while Swaziland, Rwanda and Zambia have developed draft policies, subject to approval by their respective Health Ministries. Only five countries across Africa have palliative care integrated in the curriculum of health professionals, two of the countries (Uganda and South Africa) have recognized palliative care as an examinable subject [6]. This has caused a shortage of health care professionals [7] specialized in palliative care, leaving end-of-life care service to advocacy groups and palliative care associations. Despite the initiative of the Ugandan government, care provision remains disintegrated in national public health agendas and systems, and it continues to be heavily reliant on non-governmental, community-based and home-based care (HBC) models.

In Uganda, the Ministry of Health together with Palliative Care Teams are scaling up the provision of palliative care services. This has been done through the introduction of free oral morphine as an essential medication for pain and symptom management in Uganda. However, due to financial constraints, strategies to diversify their resource base locally have been adopted [10]. With this milestone in palliative care provision, there are numerous challenges due to poor financing by the Ugandan government [3, 9]. Thus, there is need to scale up service provision, training, improve access and ensure sustainability by emphasizing revision of available policy, drug availability, education and integration of service delivery.

## Methods

### Design and rationale

A descriptive retrospective survey was adopted and a structured questionnaire was used to gather data at the 15 stand-alone (operating independently and specializing in palliative medicine) hospice/palliative care facilities on the trends of financing palliative care. A content analysis of audited accounts was undertaken. This was done using a list of all the registered palliative care providers obtained from the Palliative Care Association of Uganda (PCAU), mandated to keep a directory of all palliative care organizations in Uganda.

### Sampling procedures

A list of all registered stand-alone palliative care providers was obtained from the Palliative Care Association of Uganda (PCAU). It was then grouped into two: those that had operated for ≤5 years and > 5 years. Purposive sampling was adopted, that included the recruitment of all stand-alone palliative care providers with audited accounts in Uganda. The study included only the stand-alone palliative care services providers and hospices (those that traditionally specialize in palliative care for more than 5 years). Palliative care units operating within health facilities were excluded as it was assumed that they had no specific funds allocated for palliative care activities and palliative care is delivered as an integrated service with the health facility. Among the stand alone hospices, those which had operated for less than five years were excluded from further analysis.

### Data collection and management

A structured questionnaire developed for this study and an in-depth interview guide were used to collect detailed information of the structure, ownership, activities, financial and sustainability of the various palliative care organizations. This included certified copies of audited accounts for a period of five years. In-depth interviews were conducted with key informants with regards to issues not captured by the questionnaire and a document review was undertaken to obtain clarity of strategic issues on sustainability. Finally the data obtained from audited accounts were used to collect retrospective information on all the palliative care centres. This included documents on: profile of the organization from the management records, strategic plans, information from the management of the individual organizations, and disbursement records by government from the Ministries of Finance and Health or the host districts.

### Ethical considerations

Ethical approval was obtained from the East and Southern African Management Institute (ESAMI) and governing body of the Palliative Care Association of Uganda (Hospice Africa Uganda- *UG-REC-016)*. Written informed consent of participants was obtained.

## Results

### General findings

In Uganda, there are 15 stand-alone hospices or palliative care centres. Nine of these have operated for more than five years. Six (40%) have operated for less than five years; while nine hospices (60%) had operated for between five and 25 years, with an average of 12 years. The hospices and palliative care units are spread across all the regions of Uganda. The six hospices that had operated for less than five years were excluded from the study. Of the nine hospices left, only eight responded, giving a response rate of 89%.

#### Services provided by the hospices

The range of services offered by the palliative care providers are summarized in Table 1. Seven of the eight palliative care providers offered outpatient, training and research; two provided inpatient services. Most of the providers liaised with nearby hospitals and health centres for patients. The palliative care services were mostly home-based.

**Table 1:** Services provided by the Hospices

No.	Activity	Yes	No	Total
1	Out Patient services	7	1	8
2	In patient services	2	6	8
3	Training	7	1	8
4	Research	7	1	8

#### Categories of clients

Four of the nine palliative care providers offered palliative care in HIV/AIDS care exclusively and only three hospices provided comprehensive aspects of palliative care for all patients. One provider did not attend to patients, but coordinated referrals to other palliative care providers. The types of conditions seen in the various units are as shown in Table 2.

**Table 2:** Conditions Seen In the Various Units

No.	Condition	No.	Condition
1	Cancer	7	Kidney
2	HIV/AIDS	8	Heart Disease
3	Cancer and HIV/AIDS	9	Malaria
4	Sickle Cell Disease	10	Typhoid
5	Burns	11	Pneumonia
6	Liver Disease	12	Diarrheal Diseases

### Findings related to finance

#### General financial issues

The general financial issues included donors, financial statements, audited accounts, sustainability plans, grant offices, income generating activities and government contributions. All the palliative care providers received external donor funds, ranging from three to over 10 external funders. Further, there was a positive correlation between the years of operation and the number of funders. The summary of the financial issues is as shown in Table 3.

**Table 3:** Summary of General Financial Issues

No.	Activity	Yes	No	Total
1	Donors (3 to 10)	8	0	8
2	Approved Financial statements	8	0	8
3	Approved audited accounts	8	0	8
4	Sustainability Plan	6	2	8
5	Grants office	6	2	8
6	Income Generating activities	2	6	8
7	GOU contribution	3	5	8

#### Financial statements and audited accounts

Eight hospices that responded had financial statements for the period they have been in operation. A content analysis of the period under review showed that the accounts were found to be adequately audited by external auditors endorsed by the Boards of Directors and approved in the Annual General Meetings as specified in the Company’s Act of Uganda. The summary of the financial statements are further clarified in the analysis below**.**

#### Sustainability plans

Six of the eight (75%) stand-alone palliative care centres had sustainability plans included in the strategic plans of the organizations and outlined what they should do to ensure long term survival.

#### Grant offices

Four of the eight (50%) stand-alone palliative care centres had grants management offices, with personnel fully dedicated to grants and proposal writing. Three (34.5%) of these had full time financial mobilizers and fundraising officers. Two (25%) did not have any local grants or proposal writing activities, and relied only on funds mobilized by external supporters. The older organizations had better organized and active grants offices.

#### Income generating activities

Only three (42.9%) of the eight stand-alone palliative care centres had functional income generating activities (IGAs). The range of these activities and the number of providers are shown in Table 4.

**Table 4:** Income Generating Activities

Income generating Activities	Number
1. Membership Fees	7
2. Training Fees	5
3. Gift Shops and Flea Markets	3
4. Accommodations and Rentals	1
5. Agriculture	1
6. Sale of Beads	1
7. Baking Cakes	1
8. Ambulance Services	1
9. Others	3

#### Government of Uganda’s contribution

Three of the seven (37.5%) centres received assistance from Uganda’s government, through the Ministry of Health. The assistance was in the form of: support/provision of training, payment of taxes, and supply of medicines and availability of office space. All the service providers received free oral morphine through the Joint Medical Stores, the scope of assistance is given in Table 5.

**Table 5:** GOU Contribution

No.	Activity	Number
1	Support/provide training	6
2	Supply of medicines	6
3	Payment of taxes	3
4	PHC grants	2
5	Office space	1

#### Other activities provided by the PC organizations

During the survey, important new activities which the organizations are undertaking, which were not originally their core functions, were uncovered. New areas which the organizations diversified into are as listed in the Table 6 below. These included grant management for other NGOs, training of laboratory assistants, HIV prevention interventions, safe male circumcision, cancer screening programs, counseling and guidance as indicated in Table 6.

**Table 6:** Other activities provided by Palliative care organization

No.	Activity	Number
1.	Grant management for other NGOs	3
2.	General counseling and guidance	2
3.	Cancer screening programs	2
4.	Training of laboratory assistance	1
5.	HIV prevention services including male circumcision	1
6.	HIV care and support	1

### Analysis of the financial statements

#### Sources of funds for the palliative care providers

The stand-alone palliative care providers had two sources of income: 1) Donations (local and international grants) and 2) Income generating activities from axillary projects, provide training in short courses on palliative care, co-payment of service fee and membership fees. The total amount of funds received by all the stand-alone palliative care providers over the five year period was Ushs 123,451,035,673/=; of which Ushs 115,086,594,042/= (93.22%) was from donations and Ushs 8.364, 441,631/= (6.78%) was from income generating activities within the organizations. The percentage contribution from donations to total income ranged from 100% for Kitovu Mobile to 79.34% for Kawempe Home Care (KHC). Other centres assessed: Rays of Hope Hospice Jinja (99.05%), Palliative Care Association of Uganda (PCAU) (91.32%), Mildmay Uganda (95.34%), HAU (95.3%), and Reach Out Mbuya (88.72%). The percentage contribution from locally generated funds ranged from 20.66% for KHC, to 0.95% for Rays of Hope Hospice Jinja; with an average of 6.81%. The others were Mildmay Uganda (4.66%), HAU (10.7%), Reach Out Mbuya (11.28%) and PCAU (8.68%).

Rays of Hope Hospice Jinja had a total income of 603,375,127 UGX over the five years. Of this, 99% were donations from eight donors that is; three from UK, two from USA and one each from Ireland, the Netherlands and African Palliative Care Association (APCA), and 93% of this income is used as operational funds.

HAU had a total income of 22,346,062,751 UGX, 89% from donations and 11% from local income generating activities. There were 58 sources of funding; 7 from locally generated funds (one time or monthly cash donations, in-kind donations that is medicines and sandices, corporate social responsibilities), 33 from international donations and 16 were from local income generation activities (axillary projects, short courses in palliative acre, co-payment of user fees, retailers and membership fees). The local donors included PCAU/MPCU, MOH, Airtel, Centenary Bank, individuals and other unspecified sources. There were 33 international donors; the majority from UK, USA and Europe. The income generating activities included education fees, rentals, sale of oral morphine, bank interest and disposal of assets.

Kawempe Home Care had a total income of 1,526,079,266 UGX; of which 54% was from international donations, 25% from local donations and 21% from local income generating activities.

Palliative Care Association of Uganda had a total income of 2,314,470,196 UGX from 17 sources during the period under study; of which 12 were local donations (cash of one time or monthly donations from Ugandans), fund raising rally and four were income generating activities(short courses, membership fee). International donations constituted 91.2% of the income, internally generated funds constituted 7.7% and local donations were up to 1.1%.

Reach Out Mbuya had a total income of 11,352, 288,252 UGX from 22 sources; ten of which were local donations (6%), eleven were international donations (83%) and one income generating activity (11%).

**Mildmay Uganda (MU)** had a total income of 81,218,366,800 UGX. International donations contributed 90%, local donations and income generating activities contributed 5% each. There were seven sources of donations; three international donations, and two each local donations and income generating activities.

## Discussion

There were fifteen stand-alone hospice and palliative care providers operating in Uganda during this period. Uganda has one of the most developed palliative care services in Africa [6] as an integral component in the national health system. This study independently verified the funding of stand-alone Palliative Care organizations in Uganda. Stand-alone palliative care services have been available in Uganda for the last 25 years, covering 69 of the 112 districts. However, only 10% of the patients who need palliative care in Uganda have access to it [11]. Furthermore, the stand-alone palliative care units provide a range of services including outpatient- and inpatient services, training/education and research. This finding is in line with the understanding of models of palliative care services in Africa [5, 12, 13]. HAU demonstrated a strong palliative care model of home-based care with strong linkages to referral hospitals [14]. The most common conditions addressed by the stand-alone palliative care services were cancer and HIV/AIDS clients, however, with the projected increase in the incidence of non-communicable diseases, by up to 27% in the next 10 years [15] this is likely to change. The results show that most of the stand-alone palliative care services had external funding; proportionate to the duration of the palliative care operation. This may be ascribed to the operational credibility organizations attain over time, as evidenced by HAU, Mildmay Uganda, Reach Out Mbuya, and the Palliative Care Association of Uganda. The fact that palliative care is highly donor dependent raises sustainability concerns as the global economic slowdown may hamper funding opportunities. Palliative care has been greatly affected by the withdrawal of important palliative care funders from Africa such as the Princess of Wales Memorial Fund [6]. There is thus urgent need for alternative funding sources; for example Kawempe Home Care has set up strategic partnerships to acquire medicines and staff. Although with limited funding options, this approach is a key sustainable alternative as the organization locally raised 46% of its operating funds [16]. To ensure sustainability of local donations; most stand-alone palliative care providers have grants offices to help them solicit external funds. Also, some stand-alone palliative care providers offered other services, such as grant management, courses in counseling and guidance, and HIV prevention activities such as safe male circumcision. This could temporary bridge the financial gap. The results show that all stand-alone palliative care providers had strategic plans to ensure long term sustainability. For example, *“HAU is a model NGO offering impeccable holistic care to palliative patients and their families by strengthening the governance structures to ensuring effective leadership, financial and resource sustainability and having a competent team to deliver quality palliative care educational provision, clinical practice and support services”* [17]. To achieve this, varied approaches ranging from strong memberships to permanent partnerships with established palliative care providers in developed countries are being considered [18]. To strengthen sustainability, the APCA has intensified its promotion across Africa through integrating palliative care into national health systems. Such as the inclusion of palliative care within the basic curriculum to foster implementation [19]. Further, the approach accentuates partnership frameworks and investing in information, research and evidence gathering to ensure visibility of the palliative care services in Africa to attract funding [20, 21]. In addition, provision of technical assistance for organizational development in terms of: infrastructure, governance, financial and human resource management, strategy development and implementation, monitoring and evaluation, fund raising, marketing and branding have been considered.

To sustain their economic bases, the organizations had varied income generating activities that include; tuition fees, agriculture, bakery, ambulatory services, membership subscriptions and accommodation facilities for students and visiting scholars. However, these do not raise adequate funds fostering a need to scale up innovation. One of the challenges reported was that donor funds are geared towards supporting care and training for HIV/AIDS patients, although there are more cancer patients than AIDS patients under their care. In Uganda, there are numerous cancers on the rise and this necessitates donor support specific to cancer. The palliative care organization funds are strained because of inadequate funding to meet staff related costs. This has continued to be a challenge [18]. The government of Uganda provides funding to only three organizations, mainly in the form of medicines, training support including scholarships, and payment of taxes. Cash in terms of primary health care conditional grants were only available to Reach Out Mbuya through Kampala Capital City Authority. Rays of Hope Hospice Jinja was being provided with office space at the premises of the District Health Office. Notably, six stand-alone palliative care organizations providing patient services received free oral morphine from the Ministry of Health through the Joint Medical Stores. The government also provides essential medicines to the NGOs including those providing palliative care. This contribution by the Ministry of Health fulfills its mandate according to the WHO pillars of palliative care “to ensure integration into the national health system” [20].

### Methodological assumptions and limitations

It was assumed that all of the stand-alone palliative care providers are registered with the Palliative Care Association of Uganda. This may not always be the case, as some providers may not have registered. There may also be stand-alone palliative care providers operating in the hospitals but not registered with PCAU. This study has limitations arising from the small sample size and the fact that palliative care remains a relatively new service in Uganda.

## Conclusion

In Uganda, stand-alone palliative care services that are vital for the patients and families in need are financially constrained. The financial constraints may be minimized through an increase in domestic revenue, widening the scope of services, National Social Health Insurance and Integration of Palliative Care into the National Health System.

## Data Availability

The data is available to the public and or can be obtained from the Palliative Care Association of Uganda.

## Abbreviations

APCA: Association of Palliative Care Africa
HAU: Hospice Africa Uganda
PCAU: Palliative Care Association of Uganda
ROM: Reach Out Mbuya
